# The Convergence Between Cultural Psychology and Developmental Science: Acculturation as an Exemplar

**DOI:** 10.3389/fpsyg.2020.00887

**Published:** 2020-05-12

**Authors:** Seth J. Schwartz, Ágnes Szabó, Alan Meca, Colleen Ward, Charles R. Martinez, Cory L. Cobb, Verónica Benet-Martínez, Jennifer B. Unger, Nadina Pantea

**Affiliations:** ^1^Leonard M. Miller School of Medicine, University of Miami, Coral Gables, FL, United States; ^2^School of Health, Victoria University of Wellington, Wellington, New Zealand; ^3^Department of Psychology, Old Dominion University, Norfolk, VA, United States; ^4^College of Education, The University of Texas at Austin, Austin, TX, United States; ^5^ICREA and Universitat Pompeu Fabra, Barcelona, Spain; ^6^Keck School of Medicine, University of Southern California, Los Angeles, CA, United States; ^7^School of Psychology, Universitatea Babes̨-Bolyai, Cluj-Napoca, Romania

**Keywords:** cultural psychology, developmental science, acculturation, international migration, mutual constitution, person↔context relations

## Abstract

The present article proposes an integration between cultural psychology and developmental science. Such an integration would draw on the cultural-psychology principle of culture–psyche interactions, as well as on the developmental-science principle of person↔context relations. Our proposed integration centers on acculturation, which is inherently both cultural and developmental. Specifically, we propose that acculturation is governed by specific transactions between the individual and the cultural context, and that different types of international migrants (e.g., legal immigrants, undocumented immigrants, refugees, asylum seekers, crisis migrants) encounter quite different culture–psyche interactions and person↔context relations. We outline the ways in which various acculturation-related phenomena, such as acculturation operating at macro-level versus micro-level time scales, can be viewed through cultural and developmental lenses. The article concludes with future directions in research on acculturation as an intersection of cultural and developmental processes.

## Introduction

Cultural psychology has been well established as a scientific discipline for several decades. Although the term *‘cultural psychology’* was first introduced by DeVos and Hippler in 1969, its theoretical and historical roots go as far back as the 1920s, when the Vygotsky-Luria Circle, an interdisciplinary group of psychologists, physicians, and educators, was established. Their collaboration centered around the idea of an integrative psychological theory based on the premise that mind, body, and culture were inseparable and that their development was fundamentally shaped by the individual’s socio-cultural context. Although the goal of creating a unified theory never came to fruition, the legacy of the Vygotsky-Luria Circle inspired and influenced many schools of thought, including the development of the field of cultural psychology in the 1970s. Nonetheless, there remains a need to integrate developmental science principles into cultural psychology.

As such, the present article is intended to briefly review cultural psychology as a field and to integrate cultural psychology with developmental science. As we state in more detail below, cultural psychology is inherently a developmental discipline, and developmental science is inherently cultural. We use acculturation – which is defined as both a cultural and a developmental process (Berry, 2017) – as an exemplar to illustrate how cultural psychology and developmental science might be integrated. We seek to elucidate precisely *what is cultural* and *what is developmental* about acculturation and similar phenomena. We do not believe that the cultural and developmental components can (or should) be separated, but we do believe that each set of components should be enumerated. Doing so may help to pose important questions and directions for the fields of cultural and developmental science, and for the constructs that represent their intersection. We will focus primarily on international migration, given that acculturation applies largely to migrants and their immediate descendants. This article is organized into four primary sections: key postulates of cultural psychology, key postulates of developmental science, acculturation as an exemplar of the intersection of cultural psychology and developmental science, and future directions for acculturation research.

## Key Postulates of Cultural Psychology

Cultural psychology focuses primarily on understanding the ways in which cultural processes and human psychological functioning interplay and shape each other (Shweder, 1991). An important point of distinction of cultural psychology from other branches of psychology, and from cross-cultural psychology in particular, lies in its relativist approach (Heine and Ruby, 2010). Cultural psychologists do not pursue cultural comparisons with the main objective of finding universals. On the contrary, they are interested in uncovering how cultural practices or shared traditions interact to shape psychological functioning in distinctive ways. Their investigations are informed by an underlying assumption that the same cultural processes might serve different purposes in different contexts (Rogoff, 2003).

Cultural psychology posits that human experience is the product of the reciprocal interaction between culture and psyche. This premise, however, has become increasingly complex to study within diverse sociocultural contexts, where psychological functioning and human development are simultaneously influenced by multiple cultural realities. This principle is aptly illustrated by how our understanding and conceptualization of culture have evolved in response to the ethno-cultural and linguistic diversification of contemporary societies.

Culture was once commonly defined as a system of understandings shared by a group of people and described as an operating system that is “invisible and unnoticed, yet playing an extremely important role in development and operation” (Matsumoto, 2001, p. 3). However, this depiction of culture has been increasingly criticized by cultural psychologists, because it provides a rather uniform and static view of cultural processes, especially in an increasingly global and diverse era when people from different cultures come into contact on a daily basis. Morris et al. (2015) argue that the main problem with the “operating system” metaphor is the underlying assumption that multiple cultural systems cannot co-exist within an individual without difficulty. As most computers are unable to run two operating systems simultaneously, if culture was indeed like an operating system, it would be very challenging, if not impossible, for individuals to navigate and exist within multiple cultural frameworks. However, a large body of research undertaken with immigrants and ethnic minorities shows that this is not the case at all (Hong et al., 2000; Nguyen and Benet-Martínez, 2013).

Instead, Morris et al. (2015) propose that “cultural knowledge is more like a set of apps that users select or even download unwittingly in the course of exploring the web” (p. 639). Specifically, just as users open apps for specific purposes (e.g., word processing, email, statistical analyses), individuals activate cultural knowledge – purposefully or otherwise – based on the specific surroundings and contexts in which they find themselves or with which they seek to engage. Cultural knowledge or schemas as a set of “apps” can thus frame, transform, and regulate all aspects of psychological functioning when they are activated and are relevant to the situation at hand. Which apps will affect how we think, feel, and behave depends on the types of schemata and knowledge we have acquired within and across cultures through the process of acculturation.

This inherent interconnectedness is analogous to the person↔context relations postulate from developmental science (Lerner and Overton, 2008). Culture and context provide opportunities for different experiences to emerge and shape psychological processes in unique ways. Culture molds the way we see the world and think, the ways in which we relate to others, and even our biology. For example, Park and Huang (2010) review research indicating that the brain regions activated when people are asked to think about their mother differed between North American and East Asian cultural contexts – suggesting that cultural differences in the importance and role of family become imprinted in the brain. At the same time, cultural traditions, values, and practices evolve along with the changing needs of communities. Some of these changes might evolve slowly and organically over time (e.g., practices around celebrating weddings), whereas others might require more direct action (e.g., achieving marriage equality for same-sex couples). There are many examples of people successfully challenging cultural norms and initiating social change through advocacy.

In developmental science, this mutuality has also been understood within the framework of co-constructionism. Co-construction postulates that while psychological processes exist at the individual level, they have a socio-cultural origin and are constructed through interaction with the broader environmental context (Valsiner, 1996). Actions, thoughts, feelings cannot be interpreted in a vacuum; they only become meaningful through context and culture. Further, culture is not just an independent variable that affects psychological functioning or development in a unidirectional way – and neither are people passive recipients of cultural influences. Individuals play an active role in their development by constructing and reconstructing cultural processes through interactions, interpretations, and internalization. Cultural theories have long been influential to the study of human development, starting with the psycho-cultural model introduced by Whiting (1977) and its extensions into ecological-cultural approaches (Super and Harkness, 1986; Weisner, 2002), or concepts of developmental niche (Super and Harkness, 1986), zone theory (Valsiner, 1987), and micro-niche (Worthman, 2010). One common element in all of these approaches is the underlying principle that development is a result of co-construction between person and culture (Cole, 1996). A majority of the work in cultural-developmental science has been focused on understanding patterns of child development in cultural contexts. Cultural theory is seldom applied to development beyond childhood and adolescence (for an exception, see work on adult learning; Billett, 1998) and has not yet been integrated with life-course developmental theories.

A second tenet of cultural psychology emphasizes that psychological processes are culturally patterned. This relativist approach views experiences and behavior as embedded within culture, and posits that the function of psychological processes is relative to the context in which those processes occur. The relativist approach does not necessarily imply that cultural psychologists reject universals. In fact, some would argue that the cultural grounding of human experience is what is universal in psychological functioning (Markus and Kitayama, 2010). One of the most well-known examples of this grounding is the ways in which cultural practices enable the development and maintenance of independent versus interdependent self-construals (Markus and Kitayama, 1991). An independent self is rooted in personal attributes and characteristics, the unique configuration of which provides the person with a sense of individuality. Consequently, thoughts, feelings and actions are mainly determined by the person’s needs, goals, and desires. An interdependent self, on the other hand, is embedded within and defined by a network of social relationships. The self is not seen as a unique entity, and identity is derived from meaningful social connections. In this configuration of the self, thoughts, feelings and actions are mainly determined by the needs, goals, and desires of meaningful others.

Markus and Kitayama (1991, 2003) have argued that this distinction cannot simply be reduced to differences in what people value, but that such differences translate into distinctive psychological processes. They reflect different modes of being and constructing reality through engagement with cultural practices. Cross et al. (2011) reviewed 20 years of empirical research on self-construals. Although the available evidence is limited in some areas, studies converge to link independent and interdependent self-construals to specific aspects of cognition (e.g., low versus high context sensitivity), motivation (e.g., promotion versus prevention focus), emotion (e.g., wellbeing derived from self-esteem versus wellbeing derived from harmonious relationships), and behavior (e.g., direct versus indirect communication style).

A number of cultural processes are assumed to underlie variability in self-construals. Among the most prominent of these dimensions is individualism-collectivism (Triandis, 1995). Individualism encompasses cultural practices, values and traditions that promote self-reliance, self-focus, and prioritizing one’s own needs over those of family members and other close social ties. In contrast, collectivism encompasses cultural practices such as deference to family members, conceptualizing oneself as inherently connected to others, and cooperation. It is important to note that individualism and collectivism are not opposites (Oyserman et al., 2002; Taras et al., 2014). Likewise, independent and interdependent self-construals are not mutually exclusive (Markus and Kitayama, 2003). For example, people can be highly competitive at work but highly interdependent and cooperative in their family lives; or they can cooperate with others in pursuing personal goals. People living in the same context can engage in a multitude of cultural practices and daily experiences, which creates further variability and diversity within populations (Markus and Kitayama, 2010).

Although there is increasing research outside of the Western world, a major criticism of cultural psychology is its tendency to study participants with particular characteristics. Most of what we know about the interplay between culture and psychological functioning is based on studies conducted with samples in Western, educated, industrialized, rich, and democratic contexts (Arnett, 2008; Henrich et al., 2010). In addition to the range of under-represented cultural contexts, multicultural individuals (immigrants, ethno-cultural and racial minorities) are particularly under-explored. We argue that this is a missed opportunity for cultural psychology to study not only how psychological processes are shaped by multiple cultural influences simultaneously, but also the ways in which novel and hybrid cultural processes evolve from the experience of living at the intersection of cultures and contexts.

In an increasingly global world, intercultural contact has a great potential to become a central concept for cultural psychology. Within one’s own cultural (or sub-cultural) context, one’s behavior patterns may seem normative. For example, many Americans value self-enhancement and an orientation toward personal success (Bowman et al., 2009), and many Koreans and Chinese value familial honor and subjugating oneself to the needs of one’s family (Yeh and Bedford, 2004). These values, and the motivation and behaviors associated with them, may not be noticeable when one is within one’s cultural group where most people share these beliefs. Intercultural contact – communication between individuals and groups from different cultural backgrounds or contexts – brings out the cultural relativity of one’s values and behaviors. For example, Vollhardt (2010) found that, compared to Germans who had not hosted international exchange students, German individuals who had recently hosted an international exchange student were more likely to use culturally sensitive (rather than xenophobic) framing to explain the behaviors of people from other cultural contexts. Similarly, research supporting intergroup contact theory (Pettigrew et al., 2011), in which some intercultural contact research is grounded, holds that contact with people from other groups – in this case cultural groups other than one’s own – increases tolerance and decreases prejudice. A reasonable explanation for these findings is that intercultural contact increases awareness of cultural relativity (i.e., that the assumptions underlying one’s own cultural system are not the only correct assumptions, and that other cultural systems and assumptions may also be valid).

The ways in which cultural contexts frame human functioning, and the ways in which collective human action can transform cultural contexts, represent the primary areas of inquiry within cultural psychology (Adamopoulos and Lonner, 2001). However, what we learn about the embeddedness of human experience within cultural contexts is generally taken from cross-sectional surveys, descriptive and observational studies, or lab-based experiments. We know that neither culture nor human experience are static. Individuals grow and change over time, and cultural contexts evolve (Varnum and Grossmann, 2017). Matsumoto (2002) reviews the ways in which Japanese society, for example, has become increasingly individualistic since the 1970s and 1980s. Jones (2014) chronicles the political upheaval in post-Soviet Georgia following the collapse of the Soviet Union in 1991. Venezuela, once one of the wealthiest countries in Latin America, now suffers from such economic desperation that educated professionals are emigrating en masse to the United States, Spain, Italy, and neighboring Latin American countries (Tarver, 2018). Researchers studying people residing in these countries in the 1990s would be examining a different cultural context than they would had they conducted similar research in the same contexts 20 years later.

These examples highlight the need for understanding people’s *changing* lives within *changing* structural, social and cultural contexts. Toward this end, there is a need for cultural psychology to increasingly draw on life course and developmental perspectives. Integrating principles from cultural and developmental science is not a new idea. Developmental perspectives have always been ingrained within cultural psychology and, in fact, the most important contributions to theory and research in cultural psychology have come from developmental scientists, such as Barbara Rogoff, Michael Cole, Joan Miller, Patricia Greenfield, and Jaan Valsiner.

## Key Postulates of Developmental Science

Developmental science is a vast field that extends across many levels of analysis ranging from the role genes play in maturation to how the broader ecology (e.g., community and neighborhood) impacts development. Many human experiences and constructs change over the life course – from brain structure and function (DeHaan and Gunnar, 2009) to peer relationships (Rubin et al., 2009) and ethnic identification (Rivas-Drake et al., 2014). However, there are a number of developmental postulates on which we can draw to derive a developmental framework for acculturation and similar cultural/developmental constructs. These postulates include developmental systems, person↔context relations, equifinality, multifinality, and irreversibility, among others (see Lerner and Overton, 2008; Overton, 2015; Cicchetti, 2016, for reviews).

All of these properties, however, stem from a relational developmental systems (RDS; Overton, 2015) perspective that depicts human development as a property of systematic change in the multiple and integrated levels of organization that comprise development and its ecology, rather than an exclusive property of the individual or of the environment. Within this metatheoretical perspective, development can best be understood as a complex *developmental system* and emerges through *bi-directional* relationships across multiple levels of organization (e.g., biological, psychological, and social ecological levels) that are structurally and functionally integrated (Lerner, 2012).

Like other systems (e.g., biological systems, social systems), developmental systems are hypothesized to operate based on a set of lawful properties. Among these is that the various components of the system – such as person and context – mutually influence one another. Indeed, RDS rejects Cartesian polarities or false dichotomies (e.g., nature vs. nurture), including the dichotomy of *person* versus *context*. Instead, it conceptualizes the unit of development as the embodied *person-in-context* and the unit of analysis as the bidirectional relation between person and context (person ↔ context) (Gestsdóttir and Lerner, 2008). As an example, the family context shapes children’s outcomes, such that children from supportive and nurturing families generally evidence more favorable outcomes (e.g., higher self-esteem, lower depressive symptoms and risk taking behavior) compared to children from conflictual or distant families (e.g., Davis-Kean, 2005). At the same time, because the person is a co-equal contributor, a developmental systems perspective holds that the person is an active agent, rather than a passive recipient of environmental influences. As argued by Erikson (1950), Lerner et al. (2001), and Côté and Levine (2002), among others, there are important individual differences in terms of the extent to which people initiate transactions with their social and cultural environments. Individuals can draw on their own internal resources, such as agency and self-determination, to act upon their environments.

In summary, within an RDS perspective–which implies person↔context interplay – people influence, and are influenced by, their contexts. As applied to acculturation and international migration, migrants can seek out opportunities within their specific context to engage with their new cultural environment and to integrate elements of this new cultural system with their cultural heritage (e.g., Tadmor et al., 2009; Repke and Benet-Martínez, 2018; Meca et al., 2019). Migrants who adopt such an agentic and self-directed approach will likely evidence more favorable psychosocial outcomes compared to those who do not engage with the destination society or who do not retain their cultural heritage (Nguyen and Benet-Martínez, 2013; Berry, 2017). As we will note later in this article, many of the conclusions from developmental and cultural psychology are convergent and compatible.

The remaining properties of developmental systems stem from these foundational characteristics. To begin with, development is *irreversible* because the specific circumstances that contributed to a specific developmental pathway are unlikely to be undone. As a result of the vast complexity across multiple levels of organization (e.g., biological, psychological, and social ecological levels) that are structurally and functionally integrated, *equifinality* and *multifinality* represent complementary properties of conceptualizing development. Equifinality occurs when two people arrive at the same developmental milestone despite different starting points – such as one adolescent consistently achieving excellent grades in school and another adolescent becoming a high achiever despite early academic difficulties. Multifinality occurs when two people have the same starting point but evidence different change trajectories. For example, two adolescents may be exceptional students in middle school, but one of them remains a high achiever whereas the other decreases in academic achievement.

From a developmental science perspective, change and the capacity for change (i.e., plasticity) is an inherent aspect of the developing system (Lerner and Overton, 2008) – given that the system is nested within a specific and *changing* historical context. As a result, developmental processes are also *malleable*. That is, developmental scientists emphasize that interventions can be used to redirect individuals and groups onto a different trajectory (e.g., Gifford-Smith et al., 2005). Such interventions may involve changing people’s social contexts, providing individuals with new skills and competencies, or both. For example, there is a great deal of literature indicating that family strengthening programs can help to improve adolescents’ social and relational functioning and to reduce depressive symptoms, disruptive behavior problems, and obesity (e.g., Kaslow et al., 2012; Marsh et al., 2013). Similarly, programs that teach skills to children and increase contextual support (e.g., positive parent and teacher behaviors) can help to promote positive outcomes many years later, such as high school completion and gainful employment (Hawkins et al., 2005).

## The Intersection of Cultural Psychology and Developmental Science: Acculturation as an Exemplar

Although cultural psychology is based in part on developmental principles, a key feature that is missing from much cultural psychology research today is an explicit integration of *developmental theory and methodology* that enable process- and change-oriented research on cultural and psychological phenomena. Most cultural research has been either cross-sectional or experimental, and as Cohen (2010) notes, cultural processes are often inferred from the country/ies studied – such as the assumption that East Asian cultural contexts are primarily collectivist (prioritizing the group over the individual) whereas North American cultural contexts are primarily individualist (prioritizing the individual over the group).

Some developmental psychologists have called for incorporating culture into the study of human development (e.g., Miller, 2005; Causadias, 2013; Nielsen and Haun, 2015). In this article, we build on these arguments and will also pursue the reciprocal argument – that *developmental* approaches and methods need to be more explicitly incorporated into the study of *culture*. At the same time, we emphasize the important role that the integration of the study of *culture* can have on the advancement of our understanding of *developmental* phenomena. Although a wide range of constructs might be examined with the developmental study of culture and the cultural study of development, here we will focus on *acculturation*. As applied to international migration, acculturation refers to cultural adaptation occurring following immigration (e.g., language learning, acquisition of attitudes and values reflective of the destination society, and expansion of one’s sense of self to include the destination society as well as the society of origin). A Turkish person relocating to The Netherlands, for example, might learn the Dutch language and become bilingual, might adopt some individualistic Dutch values, and might come to view herself as Turkish-Dutch. Acculturation is inherently both cultural and developmental – it represents cultural change over time that occurs when two or more cultural groups (or their members) come into contact (Berry, 2017).

As a process of adaptation over time, acculturation is inherently developmental and represents an intersection between cultural psychology and developmental science (Sam and Berry, 2010). Indeed, just likely any other developmental process, acculturation emerges through bidirectional interactions between individuals and their *changing* ecology (e.g., family, peers, school.). For example, an extensive body of research has emphasized that caregivers (and other family members) undertake active efforts to socialize youth toward the values and behaviors of their own ethnic heritage (Umaña-Taylor et al., 2006, 2014; Schwartz et al., 2007). Moreover, parent–child differences in acculturation can compromise family processes and can create culturally related stress and mental health outcomes (Portes and Rumbaut, 2014; Schwartz et al., 2016).

Additionally, because acculturation is inherently a developmental process, a cross-sectional snapshot taken at any point during the process would paint an incomplete picture at best, and might mischaracterize the process at worst (Schwartz and Unger, 2017). Methodologically speaking, Maxwell and Cole (2007) review ways in which cross-sectional findings can bias the conclusions that would be reached using longitudinal designs. Theoretically speaking, cross-sectional research on acculturation may be misleading because different individuals may be at different points in the acculturation process – and as a result, we do not know whether the patterns observed represent individual differences in *timing* (e.g., one individual is further along in the acculturation process, but the other individual will catch up later) or in *approach* (e.g., the two individuals being compared have adopted qualitatively different styles of acculturation, and these style differences would continue to be observed over time).

One clear observation we can make is that the “worldviews” of developmental and cultural psychology – at least as these fields bear on the study of migration and acculturation – are quite different. Across multiple levels of organization (e.g., biological, psychological, and social ecological levels) that are structurally and functionally integrated, developmental science is concerned primarily with change processes, change patterns, and ways to intervene to redirect change processes so as to produce more adaptive or favorable outcomes. Cultural psychology focuses on the ways in which cultural processes shape cognition, motivation, emotion, and behavior, and the conditions that lead to different patterns of functioning across contexts.

Despite these differences between fields, there may be important points of convergence that can be leveraged to devise an integrative perspective on migration and acculturation. As noted at the beginning of this article, with regard to migration, acculturation represents an important point of confluence between cultural and developmental science. Acculturation is inherently a cultural construct because it refers to an interplay between culture and person – i.e., the dynamics that result when migrants and members of destination cultural groups come into contact with one another (Brown and Zagefka, 2011). It is also intrinsically a developmental phenomenon because it refers to changes in individuals’ and groups’ cultural behaviors, values, and identifications over time (Sam and Berry, 2010) – and these changes impact a variety of developmental processes and outcomes.

### Acculturation Theory and Research

Although multiple perspectives on acculturation have been proposed, Berry’s (2017) approach has been the most prominent within psychology and related fields. Berry conceptualized heritage-culture retention and destination-culture acquisition as the dimensions underlying acculturation. He proposed four acculturation orientations: separated (retains the heritage culture and rejects the destination culture), assimilated (discards the heritage culture and acquires the destination culture), integrated/bicultural (retains the heritage culture and acquires the destination culture), and marginalized (discards the heritage culture and rejects the destination culture). Berry’s model has largely been validated in cross-sectional studies (e.g., Chia and Costigan, 2006; Schwartz and Zamboanga, 2008; Des Rosiers et al., 2013) – the separated and assimilated categories have emerged, along with multiple variants of biculturalism. In those studies where the marginalized category has emerged, it has represented an extremely small segment of the sample.

In keeping with acculturation as a cultural and developmental process, some studies have also tested Berry’s model *longitudinally*. These studies were less likely to identify all four of the hypothesized categories. In a sample of Mexican American juvenile offenders in Arizona, Knight et al. (2009) extracted latent growth trajectory classes representing bilingualism (linguistic biculturalism), primarily English speakers, and monolingual English speakers (where the second and third categories represent variants of linguistic assimilation). Matsunaga et al. (2010), using measures of language use (English and Spanish) and ethnic identification among a sample of Mexican Americans, identified four latent profiles over time – three of which represented forms of biculturalism and one of which appeared to resemble assimilation.

### Extensions of Berry’s Model

A number of extensions of Berry’s acculturation model have been proposed. These models help to flesh out the specific cultural and developmental processes and contents that intersect under the auspices of acculturation – as well as on the inherent complexity involved in these intersections. For example, whereas Berry was largely silent on the specific areas in which acculturation occurs, Schwartz et al. (2010) delineated three specific content domains in which acculturation processes could be assumed to operate. These domains were practices, values, and identifications. Schwartz et al. (2010) reviewed evidence indicating that behavioral acculturation (e.g., language acquisition and retention), individualist and collectivist values, and ethnic and national identity may or may not overlap. Portes and Rumbaut (2014), for instance, provide several examples of Asian immigrant adolescents who have lost (or never acquired) proficiency in their families’ native languages but who nonetheless endorse strong heritage-cultural identities and endorse Asian values (e.g., filial piety, deference to parents, saving face). In an empirical examination, Lee et al. (2020) found that, among a sample of recently immigrated Hispanic adolescents in Miami and Los Angeles, practices tended to change first, followed by identifications and then values.

Of course, given the idiosyncratic nature of culture–psyche and person↔context relations, destination-society individuals will likely adopt their own specific and idiosyncratic reactions to migrants and migrant cultures. Haugen and Kunst (2017), for example, examined acculturation to migrant cultures among a sample of Norwegians. These authors extracted integrated, separated, and undifferentiated clusters, where integrated individuals were eager to engage with migrants, separated individuals tended to avoid and reject migrant cultures, and undifferentiated individuals scored midway between the other two clusters. Not surprisingly, separated individuals were most likely to perceive that Norwegian national identity was threatened by the presence of migrants. In general, non-migrant individuals who identify most strongly with their country of residence, and who adopt an essentialist view of the nation (i.e., only individuals with certain demographic profiles can be true members of the nation), tend to hold the most strongly negative attitudes toward migrants (Pehrson et al., 2009).

A third extension of Berry’s model involves demarcating between public and private acculturation (Arends-Tóth and van de Vijver, 2007). Such demarcation is important because the culture–psyche and person↔context relations that govern interactions with others in public settings are likely quite different from those that govern interactions within private settings. Migrants may adopt destination-cultural practices at work or school but engage primarily in heritage-cultural behaviors at home, especially with their children (Gonzalez et al., 2016). There are established literatures on cultural frame switching and cultural mixing, focusing on the ways in which children from migrant families are exposed to their heritage and destination cultural systems at home (e.g., Martin and Shao, 2016). There are, however, developmental distinctions in the ways in which these cultural frame switching and mixing processes operate (Portes and Rumbaut, 2014): for people who migrated as *older adolescents or adults*, there is often a sharp demarcation between the cultural expressions used in private versus in public. However, for people who migrated *as children*, or who were born in the destination country, the demarcation between how one behaves in public versus private spheres may be far less apparent. Indeed, it is possible that individuals raised in both cultural contexts (e.g., heritage at home and destination in public) may switch effortlessly, and frequently, between these cultural systems within a variety of social contexts.

### Developmental Understandings of Acculturation

Developmental extensions of Berry’s (2017) work make clear that acculturation is far more nuanced and complex than the original Berry model would suggest. Specifically, acculturation is likely to be regulated by person↔context interplay and by wider social-ecological influences (Meca et al., 2019), as developmental scientists (e.g., Lerner and Overton, 2008) would suggest. From an integrated developmental/cultural psychology perspective, the ways in which individual people and groups acculturate are multicausal – based on interactions between migrants and destination-society individuals *as well as* on social-ecological contexts such as family, neighborhood, political climate, and historical receptivity of the destination society toward migrants (see Fuller and García Coll, 2010, for a review). Acculturation also *affects* other developmental processes, including personal identity (Meca et al., 2017a, b) and family functioning (Schwartz et al., 2015). Further, the social-ecological conditions that guide acculturation can (and often do) change over chronological and generational time – such that the receiving context in a given country or region is likely different following a change in government, as economic conditions shift, and as political movements gain or erode rights and recognition for various segments of the population (Meca et al., 2019). Contexts of reception also likely change as the children and grandchildren of previous waves of migrants become part of the destination cultural group (van Oudenhoven and Ward, 2013).

Within a given cultural context and time, there is a great deal of diversity in the ways in which migrants acculturate. Within the same socio-cultural context, some migrants may engage more with the destination cultural system, and others may engage less. A similar statement can be made regarding retention of migrants’ cultural heritage. One trend that has been reported across a range of studies and contexts is that *young* migrants – children, adolescents, and young adults – are especially likely to be bicultural (e.g., Berry et al., 2006; Chia and Costigan, 2006; Schwartz and Zamboanga, 2008; Nguyen and Benet-Martínez, 2013). Young migrants likely engage strongly with both their heritage and destination cultural systems, because they likely have been educated in the destination society *and* likely have older family members (e.g., parents, aunts/uncles, grandparents) who are oriented primarily toward the family’s cultural heritage. Further, acculturation levels and profiles change over both “macro” (months and years) and “micro” (days and weeks) spans of time. Although research on acculturation at the micro level is only beginning, it appears that micro and macro level acculturation processes may be characterized by different patterns and correlates. Indeed, a key principle of developmental science is that the same process can operate differently across different time scales (e.g., days and weeks versus months and years; Lichtwarck-Aschoff et al., 2008; Lerner et al., 2009).

Schwartz et al. (2015, 2019) conducted studies both at the macro level (6-month time intervals) and micro level (daily intervals). At the macro level, Schwartz et al. (2015) found that, among a sample of recently arrived Hispanic adolescents in Miami and Los Angeles, acculturative change tended to be characterized by increases in Hispanic and US practices, values, and/or identifications. Youth who increased on all six acculturative components across time reported the most favorable psychological functioning (self-esteem, optimism, low depressive symptoms) and relationships with parents. Findings at the micro level (Schwartz et al., under review) with Hispanic college students in Miami indicated that daily changes were characterized as fluctuations (i.e., movement both up and down across days) in all six acculturative components, and that these fluctuations – especially fluctuations in US national identity and in collectivist values – were most deleterious for well-being, internalizing symptoms, and externalizing problems at the end of the 12-day study period.

It can therefore be surmised that, across longer periods of time (i.e., months or years), increases (or decreases) in indices of acculturation can be expected, and especially among migrant youth, increases in acculturation indices may be associated with positive well-being. Such change patterns may be most likely to take the form of biculturalism for migrant children, adolescents, and young adults – age groups who are apt to be exposed considerably to both private (heritage) and public (destination) cultural systems. Across short periods of time, however – such as days or weeks – change in acculturative processes are more likely to lead to negative psychosocial outcomes. One conclusion that can be drawn is that gradual change in acculturative processes may be most adaptive, and that sharp or sudden changes or fluctuations may be destabilizing and upsetting. It is possible that the person↔context relations that drive longer-term changes in acculturation are more stable, whereas the person↔context relations that drive daily changes are less so.

### Types of Migrants

Contemporary theories of acculturation stipulate that acculturative processes – and therefore culture–psyche interactions and person↔context relations – operate quite differently across various types of migrants. Steiner (2009); Berry (2017), and Salas-Wright and Schwartz (2019) have enumerated several migrant types – namely legal immigrants, undocumented immigrants, refugees, and crisis migrants. Briefly, legal immigrants move by choice and with valid documentation for long-term stay in the destination country; undocumented immigrants cross national borders illegally or overstay their visa; refugees are displaced by wars, natural disasters, despotic governments, famines, droughts, and other catastrophic events and are involuntary settled in other countries by international aid agencies; asylum seekers move voluntarily but under duress, and request emergency permission to enter or remain in a new country; and crisis migrants do not fit neatly within any of the other categories. Some may seek asylum, whereas others immigrate illegally and still others may classify as refugees. The Syrian migration to Europe and the Venezuelan migration to the United States and to other South American countries represent examples of crisis migration. As Salas-Wright and Schwartz (2019) note, crisis migration is characterized by a number of readily recognizable features: (a) the move is unplanned or hastily planned; (b) the flow of migrants is large, and many migrants arrive in the destination countries within a relatively short time span; (c) the migrant flow originates from a single country or region, but migrants settle in a variety of destinations (usually the nations that are willing to accept them); and (d) migrants arrive with considerable traumatic exposure and mental health burdens. It stands to reason that these various migrant types would experience quite different culture–psyche interactions and person↔context relations. We discuss some of these differences in the next section, where we propose ways to incorporate fundamental cultural-psychology and developmental-science principles into acculturation theory and research.

## Incorporating Key Developmental and Cultural Principles Into Acculturation Research

Given the inherently developmental operationalization of acculturation, longitudinal studies are necessary to identify the specific patterns of, and challenges to, acculturation and adaptation within and across the migrant types enumerated in the previous section. It is also essential to conduct such studies with sufficient statistical power and nuanced measures to facilitate differentiation between legal and undocumented migrants, differentiation between refugees and asylum seekers, and identifying and separating crisis migrants from other migrant types. Thus far, the confluence between cultural and developmental science perspectives on acculturation has been limited largely to cross-sectional and experimental studies, which do not permit examination of how acculturative processes change over time in response to interactions between migrants and destination-society individuals, and in response to the social-ecological factors that are related to (and may be a function of) these interactions.

It is essential for such longitudinal work on acculturation to incorporate developmental principles such as equifinality, multifinality, and person↔context relations (Meca et al., 2019). The cultural principle of mutual constitution appears to be consistent with these developmental science themes. For example, two Turkish migrants in The Netherlands may start with the same levels of Turkish and Dutch practices, values, and identifications, but these two individuals may deviate considerably in their over-time trajectories of these acculturation components. These individual differences may be rooted in the two people’s families, in the communities where they settle, or in the environments where they find employment.

It is also essential for developmentally oriented work in acculturation to incorporate cultural principles. Examining how cultural realities, such as the prevailing value systems and sets of acceptable behaviors and interaction styles, influence person↔context relations among migrants is an important research direction. It is important to examine how the cultural-developmental interplay may manifest differently for different categories of migrants – and especially (a) between legal and undocumented immigrants and (b) between “unwanted” or “threatening” migrants (e.g., crisis migrants, undocumented immigrants, and refugees) and other types of migrants. It is possible, for example, that undocumented immigrants may be scorned if they attempt to publicly identify with the nation where they reside (Staerklé et al., 2010). Legally admitted immigrants, on the other hand, may be *encouraged* to identify publicly with their nation of residence (Kessler et al., 2010). Unwanted or threatening migrants may be deliberately excluded or rejected from the destination society, as well as blamed for that society’s social ills and problems (Chavez, 2013). As a result, the opportunities available to undocumented immigrants and to some refugee and crisis migrant groups may be limited by constraints imposed by the destination society.

### Need for Pre-migration Timepoints

There may also be important *pre-migration* differences across migrant individuals, and these pre-migration differences may elicit specific culture–psyche interactions and person↔context relations. For example, Turkish migrants in The Netherlands may have lived in different parts of Turkey, may have interacted differently with Turkish social institutions, or may have left behind more versus fewer social ties in Turkey. Indeed, incorporation of pre-migration timepoints into longitudinal studies of acculturation is an important future direction (Tartakovsky, 2009; Salas-Wright and Schwartz, 2019). A *de facto* assumption in much migration research is that all migrants from a given group are equivalent upon arrival, or that experiences occurring prior to migration are not important. However, Tartakovsky’s work, conducted with Russian and Ukrainian Jewish adolescents and young adults planning to move to Israel, indicates that pre-migration ethnic identity predicts post-migration perceived discrimination. That is, youth who were more attached to Russia or Ukraine prior to moving to Israel were most likely to perceive themselves as being discriminated against once they were living in Israel. So pre-migration experiences may contribute to equifinality and multifinality in acculturation (and other migration-related experiences) following migration. Pre-migration experiences may also be part of the person↔context relations that direct developmental processes.

Assessment of pre-migration experiences may be of greatest importance vis-à-vis refugees and crisis migrants. By definition, refugees and crisis migrants have experienced traumatic conditions that led them to leave their homelands suddenly (or to be forcibly displaced). As an example, Scaramutti et al. (2019) surveyed a sample of adult Puerto Rican Hurricane Maria survivors – half of whom had relocated to Florida following the storm and half of whom had remained on the island. These authors found that more than 65% of individuals who had migrated to Florida met clinical criteria for post-traumatic stress disorder. Although Puerto Ricans are US citizens, their experiences migrating to the US mainland are similar to those of other Hispanic migrants (Acosta-Belén and Santiago, 2018). Studies tracking the acculturation of these hurricane migrants would have to consider these people’s traumatic exposure – experiencing a strong Category 4 hurricane with sustained winds of 155 miles per hour (248 km per hour), losing their homes and many of their possessions, and making a hurried and unplanned move to the US mainland. It is likely that similar statements could be made about Syrians and Venezuelans fleeing crumbling societies, Central Americans fleeing gang violence, and individuals fleeing civil wars in various African nations. Although it is logistically difficult to assess people before they migrate, doing so would help us understand the person↔context relations, equifinality, and multifinality that these migrants experience following arrival in their destination societies.

### Longitudinal Changes in Public Versus Private Acculturation

Yet another direction for future developmental research on migration involves the ways in which migrants express their heritage and destination cultural “selves” in private versus public settings. That is, are trajectories of acculturative processes different in terms of how migrants interact with family members and close friends versus how they operate in public settings such as work and school? Arends-Tóth and van de Vijver’s (2007) demarcation of public versus private acculturation focused on language use and other cultural practices, but do migrants also identify with their heritage and destination societies differently when they are at home than when they are at work or school? Do expressions of individualist and collectivist values differ across public versus private settings? Do the developmental trajectories of these public versus private types of acculturation predict psychosocial and health outcomes differently? How are such differences attributable to culture–psyche interactions and person↔context relations?

It is also important to examine the extent to which daily fluctuations in acculturative processes – which have been shown to negatively predict mental health (Schwartz et al., 2019) – may be a consequence of discomfort with having to change one’s cultural self between public and private settings. Given Rudmin’s (2003) postulate that greater distance between heritage and destination cultural systems is more likely to lead to difficulties with acculturation and biculturalism, can a similar statement be advanced regarding the cultural distance between one’s private and public cultural contexts? How does such cultural distance predict difficulties with acculturation, and does this predictive effect differ across migrant types?

## Conclusion

In this article, we have outlined both cultural and developmental approaches to the study of international migrants, and have focused on acculturation as a point of confluence between cultural and developmental perspectives. The cultural context for migrants’ developmental trajectories of acculturation (and other cultural processes) is framed by the interplay between migrants and destination-society individuals – and this interplay and the contexts that it creates are more versus less supportive for some migrant groups than for others. There are also important individual differences *within* migrant groups in terms of cultural adjustment – differences that may be due, at least in part, to experiences occurring prior to migration, as well as different interactions with family members, peers, coworkers, neighbors, et cetera.

It is also essential to capitalize on the information synthesized here to design interventions to promote successful acculturation among migrants *as well as* to increase destination-society individuals’ receptivity to migrants. To be most effective, such interventions should involve both cultural and developmental principles. For example, facilitating contact between migrants and destination-society individuals may help to bring migrants and destination society members closer together and change the person↔context relations for both groups. At the same time, it is also essential to consider where individual migrants stand in terms of their pre-migration experiences, their interactions with social contexts within the destination society, and their specific acculturation profiles. Indeed, approaches have been developed that allow for specific intervention components to be delivered to individuals and subgroups who most need them (Collins et al., 2014). It is our hope that the present article will help to advance the study of international migration and to facilitate the design of interventions to help migrants to thrive within their destination societies and communities.

## Author Contributions

SS drafted the initial manuscript. ÁS, AM, VB-M, and CW reframed the cultural and developmental sections of the manuscript. CM, CC, JU, and NP made other substantive edits and comments.

## Conflict of Interest

The authors declare that the research was conducted in the absence of any commercial or financial relationships that could be construed as a potential conflict of interest.
